# Favipiravir Inhibits Zika Virus (ZIKV) Replication in HeLa Cells by Altering Viral Infectivity

**DOI:** 10.3390/microorganisms11051097

**Published:** 2023-04-22

**Authors:** Evelyn J. Franco, Kaley C. Hanrahan, Ashley N. Brown

**Affiliations:** 1 Institute for Therapeutic Innovation, Department of Medicine, University of Florida College of Medicine, Orlando, FL 32827, USA; e.franco@ufl.edu (E.J.F.); kaley.hanrahan@medicine.ufl.edu (K.C.H.); 2 Department of Pharmaceutics, University of Florida College of Pharmacy, Orlando, FL 32827, USA

**Keywords:** favipiravir, Zika virus, antiviral therapy, infectivity

## Abstract

This study aims to evaluate the antiviral potential of the nucleoside analogue favipiravir (FAV) against ZIKV, an arbovirus for which there are no approved antiviral therapies, in three human-derived cell lines. HeLa (cervical), SK-N-MC (neuronal), and HUH-7 (liver) cells were infected with ZIKV and exposed to different concentrations of FAV. Viral supernatant was sampled daily, and infectious viral burden was quantified by plaque assay. Changes in ZIKV infectivity were quantified by calculating specific infectivity. FAV-related toxicities were also assessed for each cell line in both infected and uninfected cells. Our results demonstrate that FAV activity was most pronounced in HeLa cells, as substantial declines in infectious titers and viral infectivity were observed in this cell type. The decline in infectious virus occurred in an exposure-dependent manner and was more pronounced as FAV exposure times increased. Additionally, toxicity studies showed that FAV was not toxic to any of the three cell lines and, surprisingly, caused substantial improvements in the viability of infected HeLa cells. Although SK-N-MC and HUH-7 cells were susceptible to FAV’s anti-ZIKV activity, similar effects on viral infectivity and improvements in cell viability with therapy were not observed. These results indicate that FAV’s ability to substantially alter viral infectivity is host cell specific and suggest that the robust antiviral effect observed in HeLa cells is mediated through drug-induced losses of viral infectivity.

## 1. Introduction

Zika virus (ZIKV) is a mosquito-borne flavivirus endemic to Africa and Asia, that poses a substantial threat to global health as a consequence of severe complications associated with infection. Prior to the first reported outbreak in 2007 [1], ZIKV remained confined to endemic areas, and reports of infection were rare [2]. Outbreaks in 2013/2014 and 2015/2016 resulted in Zika’s emergence and swift spread throughout the Pacific and the Americas, respectively [3,4].

ZIKV commonly causes a self-limiting illness characterized by fever, arthralgia, rash, and headache [1,5,6]. However, more severe complications have also been described. In adults, infection has been linked to neurologic effects such as Guillain–Barré syndrome (GBS) [7,8,9]. The consequences of infection can be especially concerning during pregnancy, as there is a higher risk of preterm birth and fetal loss [10,11] among infected women. Additionally, children born to mothers infected during pregnancy are at increased risk of microcephaly, developmental delay and other congenital birth defects [8,12].

There are currently no approved antiviral therapies or vaccines available against ZIKV [9], and current treatment recommendations focus on management of symptoms through supportive care measures [13]. Due to the risk of severe, long-term consequences as a result of infection in both adults and children, it is imperative to identify effective treatment strategies against ZIKV. Antivirals specifically targeting Zika virus have not been developed; however, a promising strategy to quickly identify potential antiviral candidates is through the repurposing of existing antivirals. Favipiravir (FAV) is a broad-spectrum antiviral that is an attractive candidate for repurposing due to its oral availability and favorable side-effect profile. FAV is a nucleoside polymerase inhibitor licensed in Japan for the treatment of influenza virus [14]. It has also demonstrated antiviral activity against multiple RNA viruses [15,16], having been investigated for its activity against Ebola virus (EBOV) during the 2014 outbreak in Guinea [17]. FAV is administered as an oral prodrug that is taken up by the host cell and phosphorylated into the active moiety favipiravir ribofuranosyl-5′-triphosphate (FAV-RTP) by host cell kinases [18]. FAV-RTP acts as an analogue of endogenous purine ribonucleotides that is incorporated into the viral genome. Incorporation of FAV-RTP is thought to inhibit viral replication through activity as either a mutagen or a chain terminator [14,15].

Studies conducted by others have demonstrated that ZIKV exhibits a broad tissue tropism both in vitro and in vivo [19,20,21]. Because it is known that ZIKV can target multiple tissue types during human infection [19], and since our prior work with FAV has demonstrated its antiviral effect is variable depending on the host cell line [22,23,24], we chose to conduct these studies in three human-derived cell lines, two of which (HeLa and SK-N-MC cells) were selected as representatives of human target tissues. HeLa (human cervical adenocarcinoma) cells were utilized in these studies because ZIKV has been shown to target and persist in tissues of the reproductive tract, and reports of sexual transmission have been documented [25,26]. SK-N-MC cells, a human neuroepithelioma cell line, were selected because infection with ZIKV is associated with devastating neurologic complications [7,27]. We also evaluated FAV for its antiviral effect against ZIKV in HUH-7 (human hepatocellular carcinoma) cells. Although the liver is not considered a target tissue during infection with ZIKV, we selected this cell line since HUH-7 cells are highly permissive to ZIKV infection and have been used extensively for the study of Zika virus [28,29,30,31].

## 2. Materials and Methods

### 2.1. Cell Lines

HeLa (ATCC CCL-2) cells were maintained in Eagle’s minimum essential medium (MEM; Corning Cellgro; Manassas, VA, USA) supplemented with 10% fetal bovine serum (FBS; Sigma Aldrich; St. Louis, MO, USA), and 1% Penicillin-Streptomycin solution (HyClone; Logan, UT, USA). SK-N-MC (ATCC HTB-10) cells were maintained in MEM (Corning Cellgro; Manassas, VA, USA) supplemented with 10% FBS (Sigma Aldrich; St. Louis, MO, USA), 1% Penicillin-Streptomycin solution (Hyclone; Logan, UT, USA), 1% sodium pyruvate (Hyclone; Logan, UT, USA), and 1% non-essential amino acids solution (Hyclone; Logan, UT, USA). HUH-7 cells were maintained in Dulbecco’s Modified Eagle’s Medium (DMEM; Corning Cellgro; Manassas, VA, USA) supplemented with 5% FBS (Sigma Aldrich; St. Louis, MO, USA), and 1% Penicillin-Streptomycin solution (HyClone; Logan, UT, USA). Infectious titers were quantified by plaque assay on Vero cells (ATCC CCL-81) which were maintained in MEM (Corning Cellgro; Manassas, VA, USA) supplemented with 5% FBS (Sigma Aldrich; St. Louis, MO, USA), and 1% Penicillin-Streptomycin solution (Hyclone; Logan, UT, USA). Cells were incubated at 37 °C and 5% CO_2_, and they were split twice weekly to maintain subconfluency.

### 2.2. Virus

The 2015 human ZIKV Puerto Rican strain PRVABC59 was obtained from Biodefence and Emerging Infections Research Resources Repository (BEI Resources; Manassas, VA, USA). Viral stocks were prepared as previously described [32].

### 2.3. Antivirals

FAV was acquired from MedKoo Biosciences Inc. (Morrisville, NC, USA) and stored according to manufacturer instructions. FAV stocks of 10 mM were reconstituted in 100% dimethyl sulfoxide (DMSO).

### 2.4. Antiviral Evaluations

HeLa, SK-N-MC, and HUH-7 cells were plated onto six-well plates at a cell density of 1 × 10^6^ cells/well and incubated overnight at 37 °C, 5% CO_2_. Cells were infected at varying multiplicities of infection (MOIs) to account for differences in permissiveness to infection and to maintain comparable viral replication kinetics between cell lines. Monolayers were infected with ZIKV at an MOI of 1 for HeLa and SK-N-MC cells and 0.1 for HUH-7 cells. Virus was allowed to adsorb onto cells for 1 h, then viral inoculum was removed from each well, and wells were washed twice with phosphate-buffered saline (PBS) to remove unbound virus. An amount of 3 mL of drug containing medium at FAV concentrations ranging from 0 to 1000 µM was added to wells. Plates were incubated at 37 °C, 5% CO_2_. Viral supernatants were sampled over varying lengths of time depending on viral replication kinetics in each cell line. Supernatant samples were collected over a period of 5 days for HeLa and SK-N-MC cells and 3 days for HUH-7 cells, clarified by high speed centrifugation, and stored at −80 °C until the end of the study. Infectious viral burden was quantified by plaque assay on Vero cells.

### 2.5. Plaque Assay

Vero cells were seeded onto six-well plates and allowed to attach overnight. The following day, viral supernatant samples were serially diluted 10-fold in MEM supplemented with 2% FBS. An amount of 100 µL of each dilution was inoculated onto confluent Vero cell monolayers and incubated at 37 °C, 5% CO_2_, for 1 h. Following the 1-h incubation period, 3 mL of a primary MEM agar overlay containing a final concentration of 0.6% agar and 5% FBS was added to each well. Three days later, a secondary MEM agar overlay composed of 1% agar, 1% FBS, 200 µg/mL DEAE-dextran and 0.008% neutral red was added to each well. Plaques were counted 24 h after addition of the secondary overlay. Infectious viral burden is reported as plaque-forming units per milliliter (PFU/mL).

### 2.6. Cell Viability Assay

FAV effect on cell proliferation and viability of infected and uninfected cells was measured using the commercially available WST-1 cell proliferation assay (Roche; Mannheim, Germany). HeLa, SK-N-MC, and HUH-7 cells were seeded into 96-well plates at concentrations of 5000 cells/well for HeLa and HUH-7 cells and 25,000 cells/well for SK-N-MC cells, and they were incubated overnight at 37 °C, 5% CO_2_. The following day, medium was removed from each well, and monolayers were either mock infected with viral diluent (MEM + 2% FBS) or infected with ZIKV at an MOI of 1 for HeLa and SK-N-MC cells and 0.1 for HUH-7 cells. Monolayers were treated with different concentrations of FAV four hours after infection or mock infection. Infected and uninfected cells were incubated in the presence of FAV for three days, and then WST-1 reagent was added to each well as per manufacturer recommendations. Absorbance was measured 2–4 h after addition of WST-1 reagent using a SpectraMax M5 microplate reader (Molecular Devices; San Jose, CA, USA).

### 2.7. Quantitative Real Time RT-PCR

Viral RNA was isolated from supernatant samples using the QIAamp Viral RNA mini kit according to manufacturer specifications (Qiagen; Germantown, MD, USA), and quantitative real-time reverse transcription-PCR (qRT-PCR) was performed on extracted viral RNA samples. Viral RNA was amplified using TaqMan Fast Virus 1 Step Master Mix (Applied Biosystems; Foster City, CA, USA) and a primer-probe set targeting the 5′ untranslated region (UTR) of the ZIKV genome (Integrated DNA Technologies; Coralville, IA, USA): primers 5′-CAGACTGCGACAGTTCGAG-3′ (forward, 1000 nM) and 5′-AGAAACTCTCGYTTCCAAATCC-3′ (reverse, 1000 nM), and probe 5′-/56-FAM/CCTGTTGAT/ZEN/ACTGTTGYTAGCTYTCGCTTC/3IABkFQ/-3′ (250 nM). The thermal cycling conditions were 50 °C for 5 min and 95 °C for 20 s, followed by 40 cycles of 95 °C for 3 s and 60 °C for 30 s. Reactions were analyzed using the ViiA 7 Real Time PCR System (Applied Biosystems; Foster City, CA, USA).

Viral burden was predicted relative to the amount of viral RNA present in each sample using a standard curve generated from serial 1:3 dilutions of ZIKV stock ranging from 2.32 to 1 × 10^8^ PFU/mL. Specific infectivity was determined by calculating the ratio between infectious and predicted titers in each sample.

### 2.8. Determining Stability of Viral RNA in Tissue Culture Medium

To determine the stability of unpackaged viral RNA in tissue culture medium, viral RNA was isolated from ZIKV stock using the QIAamp viral RNA mini kit (Qiagen; Germantown, MD, USA) as per manufacturer recommendations. Then, 5 μL of viral RNA was inoculated into 45 uL aliquots of MEM (Corning Cellgro; Manassas, VA, USA) at various time points, and aliquots were incubated at 37 °C, 5% CO_2_. Viral RNA levels in each sample were quantified simultaneously at the end of the study using the qRT-PCR protocol described above, and fold changes in viral RNA levels between the 0 h time point and remaining time points were calculated using the delta C_T_ method. To determine the stability of packaged viral RNA in tissue culture medium, ZIKV stock was inoculated into MEM (Corning Cellgro; Manassas, VA, USA) to reach a concentration of 10^6^ PFU/mL. The viral suspension was incubated at 37 °C, 5% CO_2_, for up to 48 h. Viral samples were collected at various time points post-inoculation, and viral RNA was isolated from samples and quantified as described above. Fold changes in viral RNA levels relative to the 0 h time point and remaining time points were calculated using the delta C_T_ method.

### 2.9. Serial Passaging Experiment

HeLa cells were infected with ZIKV at an MOI of 1 as described above. Then, virus was propagated either in the absence or presence of FAV. Since we sought to treat infected cells with a concentration of drug that suppressed ZIKV but did not drive infectious titers below the limit of detection, a FAV concentration of 250 μM was selected for these studies. Viral supernatant samples were collected over a period of three days and frozen at −80 °C until the end of the study. On day 3 post-infection, we performed a blind passage by transferring 100 μL of viral supernatant from passage 1 onto fresh HeLa cells for passage 2. Passage 2 consisted of three experimental arms. In the first two arms, virus that was exposed to 250 μM FAV during passage 1 was propagated either (1) in the absence or (2) continued presence of 250 μM FAV (for a total of 6 days FAV exposure). Of note, residual concentrations of FAV were washed away during the infection process, since the viral inoculum from passage 1 was removed, and cell monolayers were washed twice with PBS following the 1-hour incubation period. In the third arm of the study, virus harvested from the no-treatment control arm in passage 1 was inoculated onto fresh HeLa cells and served as the no-treatment control arm for passage 2. Viral supernatant samples from passage 2 were harvested daily over a period of three days and stored at −80 °C until the end of the experiment. Infectious viral burden, viral RNA levels, and specific infectivity of these samples were determined as described above.

### 2.10. Statistical Analysis

EC_50_ values were determined by calculating the area under the viral burden time curve (AUC_VB_) for all regimens. AUC_VB_ values were graphed against the corresponding drug concentration, and an inhibitory sigmoid E_max_ model was fitted to the data using GraphPad Prism version 7.02 (GraphPad Software; La Jolla, CA, USA). CC_50_ values were calculated by graphing absorbance values at 72 h against drug concentration and fitting an inhibitory sigmoid E_max_ model to the data using GraphPad Prism software. Cell viability is reported as percent cell viability relative to an untreated control.

## 3. Results

### 3.1. Antiviral Activity of FhhAV against ZIKV

**Figure 1:**
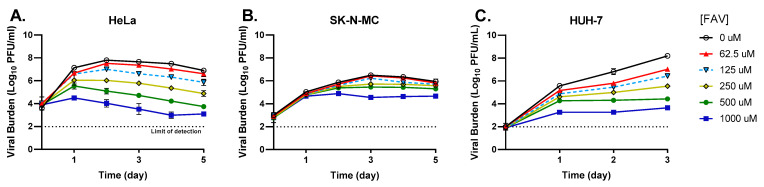
Antiviral activity of FAV against Zika virus (ZIKV) in HeLa, SK-N-MC, and HUH-7 cells. (**A**) HeLa and (**B**) SK-N-MC cells were infected with ZIKV at a multiplicity of infection (MOI) of 1, and (**C**) HUH-7 cells were infected at an MOI of 0.1. Infected cells were exposed to different concentrations of FAV. Infectious viral burden was quantified by plaque assay on Vero cells and reported as log_10_ plaque-forming units per mL (PFU/mL). Data points represent the mean of three independent samples, and error bars correspond to one standard of deviation. The dashed line signifies the assay limit of detection.

HeLa, SK-N-MC, and HUH-7 cells were all susceptible to FAV’s anti-ZIKV activity; however, the degree of antiviral effectiveness varied between cell lines. In HeLa cells, all evaluated FAV concentrations yielded declines in infectious titers that were enhanced throughout the course of therapy. For example, 500 μM FAV caused viral titers to drop relative to the no-treatment control arm by approximately 1.6 log_10_ PFU/mL on day 1, while a reduction of 3.2 log_10_ PFU/mL was achieved on day 5 (Figure 1A). FAV exhibited an EC_50_ value equivalent to 273.5 μM in HeLa cells. FAV effect was modest in SK-N-MC cells, resulting in an EC_50_ value of 388.8 μM. Low drug concentrations failed to effectively curb viral replication in this cell line, since infectious titers were comparable to the no-treatment control arm. Effective FAV concentrations (250–1000 μM) lowered peak titers by approximately 0.8 and 1.9 log_10_ PFU/mL at 250 and 1000 μM, respectively (Figure 1B). A clear dose–response effect was observed when ZIKV-infected HUH-7 cells were exposed to FAV. 62.5 μM FAV caused a 1.2 log_10_ PFU/mL reduction in peak titers, while concentrations of 250 and 1000 μM decreased titers by 2.6 and 4.5 log_10_ PFU/mL, respectively (Figure 1C). FAV achieved an EC_50_ of 218.8 μM in this cell line.

### 3.2. Cell Proliferation Assays

**Figure 2:**
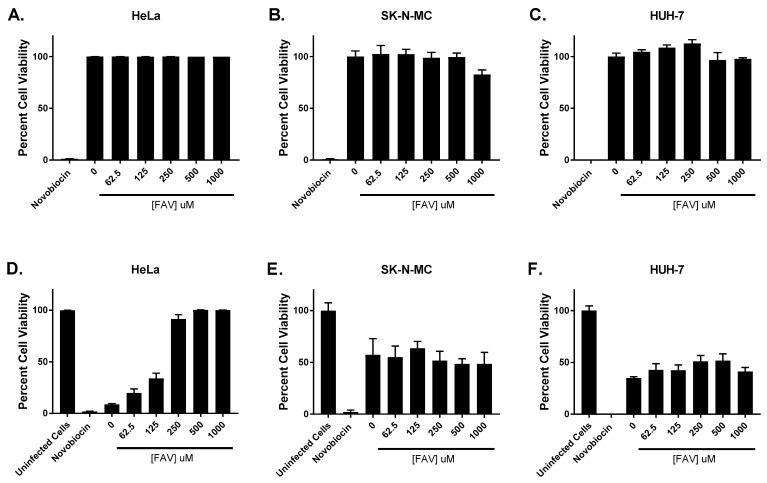
Effect of FAV exposure on viability of uninfected and infected HeLa, SK-N-MC, and HUH-7 cells. (**A**) HeLa, (**B**) SK-N-MC, and (**C**) HUH-7 cells were exposed to various concentrations of FAV. Viability of uninfected cells was measured after three days of drug exposure using the commercially available WST-1 assay kit as per manufacturer recommendations. In a separate experiment, (**D**) HeLa, (**E**) SK-N-MC, and (**F**) HUH-7 cells were infected with ZIKV at MOIs of 1 for HeLa and SK-N-MC cells and 0.1 for HUH-7 cells. Infected cells were treated with different concentrations of FAV. Cell viability of infected cells was measured after three days of drug exposure using the commercially available WST-1 assay kit. Viability of uninfected cells is reported as percent cell viability relative to the no-treatment control arm, while in infected cells, cell viability is reported as percent cell viability relative to an untreated, uninfected control. Viability assays carried out in uninfected and infected cells both used a 1 mg/mL (1575.8 μM) concentration of novobiocin as a positive control for cytotoxicity. Columns represent the mean of 6 independent replicates, and error bars correspond to one standard deviation.

Cell proliferation assays were conducted in uninfected cells to determine whether cytotoxicity contributed to FAV’s anti-ZIKV effect in each cell line. Our results indicate that FAV was not toxic to HeLa (Figure 2A), SK-N-MC (Figure 2B), or HUH-7 (Figure 2C) cells at evaluated concentrations since the mean percent cell viability remained ≥80%, and calculated CC_50_ values exceeded 1000 μM in all three cell lines. Cell viability was also measured in infected cells to determine whether FAV exposure protects cells from formation of cytopathic effect (CPE) and death. Our findings suggest FAV exerted a protective effect in HeLa cells; in the absence of treatment, infection caused a drastic decline in viability of HeLa cells; however, clear improvements in cell health were observed at all evaluated FAV exposures (Figure 2D). This observed effect was particularly pronounced at FAV concentrations ≥ 250 μM, since the percent viability at these concentrations approached that of the uninfected control arm. Infection also caused substantial declines in viability of SK-N-MC (Figure 2E) and HUH-7 cells (Figure 2F); however, unlike in HeLa cells, FAV did not exhibit the same protective effect against infection in these cell lines. In SK-N-MC cells, the percent cell viability remained constant regardless of the FAV concentration evaluated, while FAV produced only slight improvements in viability of HUH-7 cells.

### 3.3. FAV Effect on ZIKV Infectivity

Next, we sought to explain the host cell dependent differences in cell viability and antiviral activity observed in this work. We hypothesized FAV’s robust antiviral activity and protective effect against ZIKV in HeLa cells is associated with modulation of viral infectivity in the face of FAV exposure. To test this hypothesis, we measured specific infectivity over time in each cell line.

**Figure 3:**
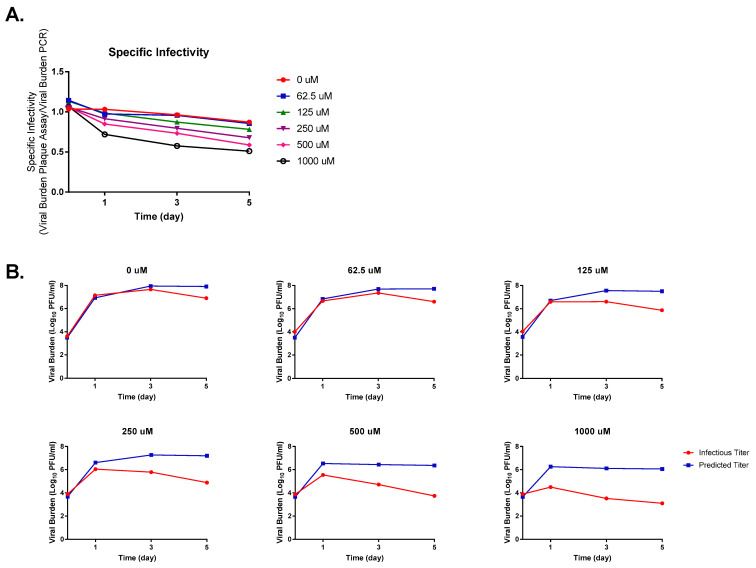
Favipiravir effect on Zika virus (ZIKV) infectivity in HeLa cells. (**A**) Changes in ZIKV infectivity were reported as specific infectivity, a ratio between infectious viral burden (quantified by plaque assay) and predicted viral burden (measured by PCR). (**B**) Comparison of infectious and predicted ZIKV titers at various FAV exposures.

The measurement of specific infectivity revealed that FAV exposure caused ZIKV infectivity to decline substantially in HeLa cells. In the absence of FAV, we detected a gradual disparity between predicted and measured titers as well as reductions in specific infectivity relative to that calculated on day 0 of up to 15.8% by the end of the experiment. When infected cells were exposed to FAV, we observed an inverse relationship between FAV concentration and viral infectivity whereby more drastic declines in infectivity were achieved as the drug concentration increased (Figure 3A). For example, on day 3, 250 μM and 1000 μM FAV caused differences between measured and predicted titers of approximately 1.5, and 2.6 log_10_ PFU/mL, respectively. The declines in specific infectivity produced by these drug concentrations relative to the control at this time point were equal to 17.39% (at 250 μM) and 40.22% (at 1000 μM). Infectivity of virus propagated under FAV exposure also decreased considerably over time in this cell line. When exposed to the clinically achievable concentration of 250 μM [22,28,33,34], comparison of levels of infectious virus and viral RNA showed there was an approximately 30-fold reduction in infectious titers on day 3, while a nearly 210-fold reduction was calculated on day 5 (Figure 3B).

**Figure 4:**
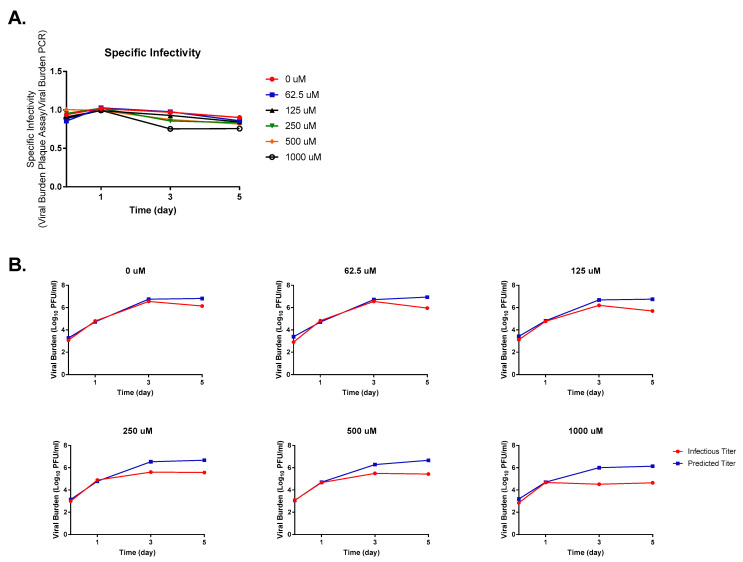
Favipiravir effect on Zika virus (ZIKV) infectivity in SK-N-MC cells. (**A**) Changes in ZIKV infectivity were reported as specific infectivity, a ratio between infectious viral burden (quantified by plaque assay) and predicted viral burden (quantified by PCR). (**B**) Comparison of infectious and predicted ZIKV titers at various FAV exposures.

Similarly to HeLa cells, FAV exposure also lessened ZIKV infectivity in SK-N-MC cells; however, drug effect on viral infectivity was not as pronounced in this cell line (Figure 4A). Decreases in specific infectivity over time were slight in the control arm, as a drop in infectivity of 4.13% was observed by day 5 of the experiment. Comparison of predicted and measured titers showed that FAV concentrations of 62.5 μM did little to change infectivity until day 5, when a nearly ten-fold reduction in infectious titers was measured (Figure 4B). Discrepancies between the levels of infectious virus and viral RNA became slightly more pronounced at FAV concentrations ≥ 250 μM, but even at 1000 μM, where the greatest decline in specific infectivity is observed, infectious titers were approximately 1.5 log_10_ PFU/mL lower than was predicted based on RNA levels (Figure 4B).

**Figure 5:**
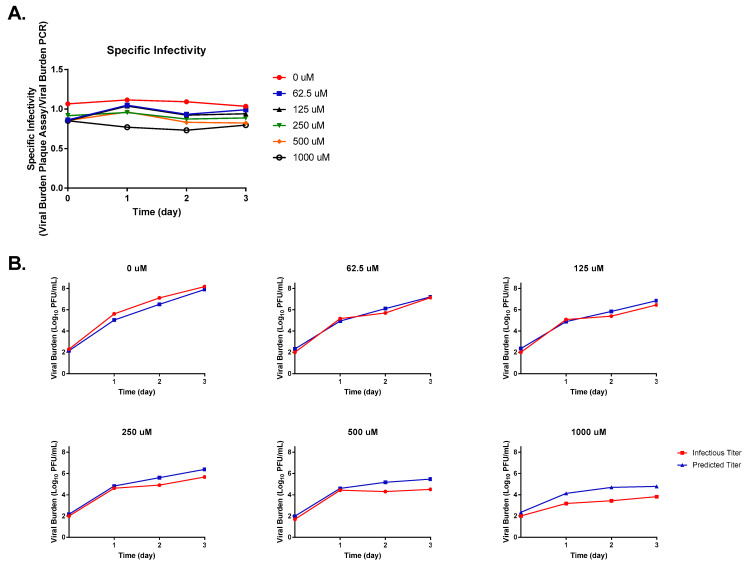
Favipiravir effect on Zika virus (ZIKV) infectivity in HUH-7 cells. (**A**) Changes in ZIKV infectivity were reported as specific infectivity, a ratio between infectious viral burden (quantified by plaque assay) and predicted viral burden (quantified by PCR). (**B**) Comparison of infectious and predicted ZIKV titers at various FAV exposures.

Comparison of predicted and observed titers in HUH-7 cells showed that RNA levels closely matched infectious titers at all evaluated FAV concentrations (Figure 5), indicating that FAV effects on viral infectivity are slight in this cell line (Figure 5A). For example, at 1000 μM, the treatment arm where the greatest discordance between amount of viral RNA and infectious virus was detected, viral titers were up to 1.26 log_10_ PFU/mL lower than was expected based on RNA levels (Figure 5B). To further illustrate FAV’s modest effect on Zika infectivity in this cell line, specific infectivity measurements showed that FAV produced declines in infectivity ranging from approximately 4.23 to 23.03% on day 3 when compared to the control arm.

**Figure 6:**
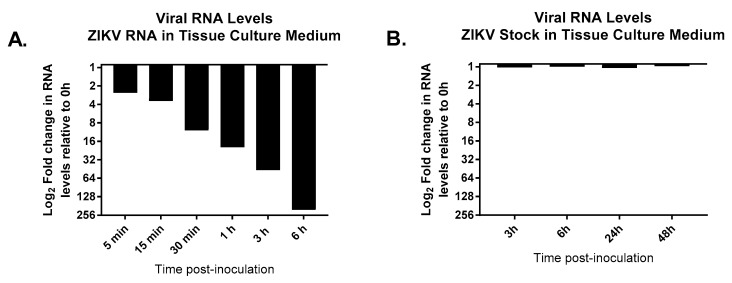
Stability of ZIKV RNA in tissue culture medium. (**A**) An amount of 5 μL of ZIKV RNA was inoculated into 45 μL aliquots of MEM at various time points and aliquots were incubated at 37 °C, 5% CO_2_. Viral RNA levels were quantified by quantitative real-time reverse transcription PCR. Changes in viral RNA levels were calculated relative to the 0 h time point using the delta C_T_ method. (**B**) A suspension containing 10^6^ PFU/mL ZIKV was generated by inoculating ZIKV stock into MEM, virus was incubated at 37 °C, 5% CO_2_, for up to 48 h. Viral samples were harvested at several time points post-inoculation, and then viral RNA was isolated from samples, and RNA levels were quantified by quantitative real-time reverse transcription PCR. Changes in viral RNA levels were quantified relative to the 0 h time point using the delta C_T_ method.

Of note, studies evaluating the stability of ZIKV RNA in tissue culture medium demonstrated that unpackaged viral RNA rapidly degrades when diluted in cell culture medium at 37 °C. For example, incubation in medium over a period of 5 min caused a nearly 2.6-fold reduction in RNA levels relative to the 0 h time point, which was used as a control, while a greater than 200-fold decrease in RNA levels was observed 6 h post-inoculation (Figure 6A). In contrast, when ZIKV stock was inoculated into tissue culture medium and incubated at 37 °C, the amount of viral RNA measured from samples collected up to 48 h post-inoculation was comparable to RNA levels measured from virus sampled at the 0 h time point (Figure 6B). These findings confirm that the extracellular viral RNA quantified in our infectivity assays was most likely isolated from RNA that was protected from degradation, likely as a result of being packaged into viral particles, rather than from an accumulation of naked RNA in the medium.

**Figure 7:**
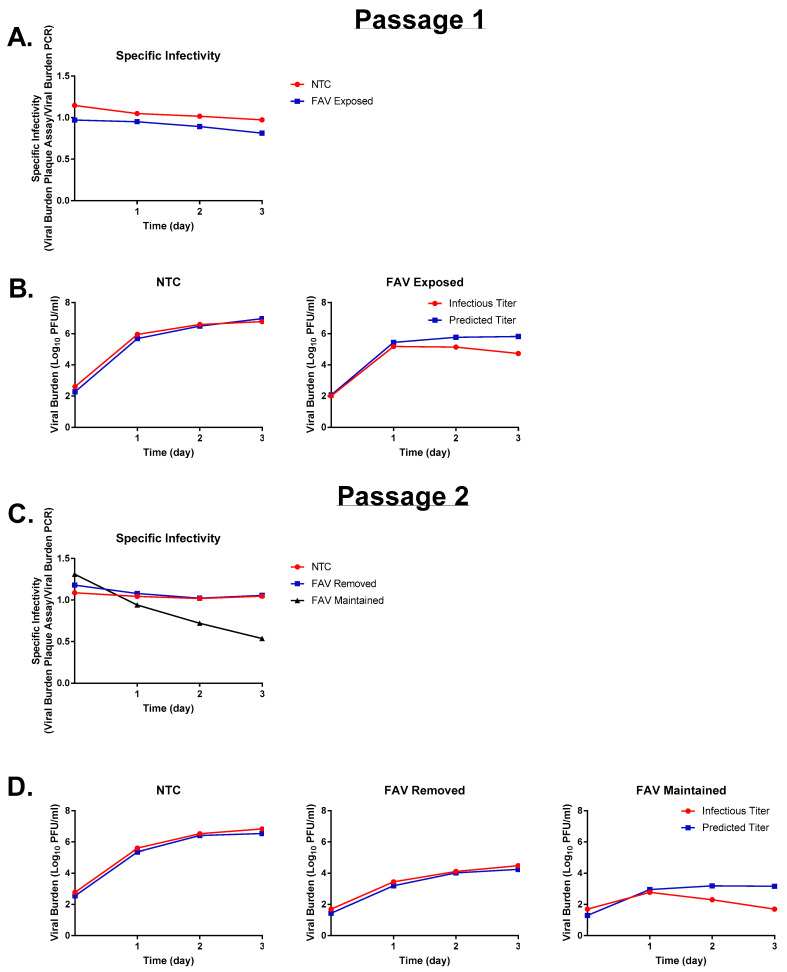
Zika virus (ZIKV) infectivity is recovered if FAV exposure is removed. (**A**) Specific infectivity of FAV exposed virus in passage 1. (**B**) Comparison of infectious and predicted titers in virus passage 1. (**C**) Specific infectivity of treatment arms where FAV exposure was either removed or maintained during passage 2. (**D**) Comparison of infectious and predicted titers in virus passage 2. Virus was passaged in HeLa cells, and no-treatment control arms were included in both viral passages.

In order to confirm that changes in viral infectivity were due to FAV’s presence, we performed a serial passaging experiment on HeLa cells to determine whether viral infectivity could be recovered following the removal of FAV exposure. During passage 1, viral infectivity of the no-treatment control arm remained constant over time (Figure 7A) since infectious titers and viral RNA levels were closely matched throughout the 3-day time course (Figure 7B). In contrast, exposure to FAV (at a concentration of 250 μM) caused a slight mismatch between measured and predicted titers that consequently led to a modest decline in specific infectivity, as was expected from previous experiments (Figure 7A,B). Among the viral samples propagated in the presence of FAV in passage 1, the removal of FAV exposure during passage 2 prompted a complete recovery of viral infectivity, as demonstrated by the lack of change in specific infectivity over time in this treatment arm (Figure 7C) and the close match between infectious titers and viral RNA levels at all time points (Figure 7D). In contrast, continued FAV exposure caused a considerable decline in specific infectivity (Figure 7C) that was particularly apparent starting on day 2 (Figure 7D). These results serve to further confirm that changes in viral infectivity observed in these experiments are dependent on the presence of FAV and that these changes can be reversed by removing FAV.

## 4. Discussion

The orally available nucleoside polymerase inhibitor FAV is a potential therapeutic candidate for the treatment of newly emerging viral infections due to its broad spectrum antiviral activity [15,16]. Previously, we have shown that FAV has potent activity against the mosquito-transmitted virus CHIKV [22,23]; however, the effectiveness of FAV is substantially influenced by the host cell line chosen for in vitro antiviral evaluations [22,23]. Here, we expanded upon our previous work by assessing FAV activity against ZIKV, an arbovirus that co-circulates with and is transmitted by the same mosquito vectors as CHIKV [35,36]. Since ZIKV replication is not confined to a single cell or tissue type [19,37,38], antiviral evaluations were carried out in three different human-derived cell lines, two of which are representatives of tissues targeted by ZIKV in man. Currently, no antiviral therapies exist for ZIKV, a circumstance that was devastating for patients, particularly pregnant patients, who were infected during the 2015–2016 ZIKV epidemic. Thus, our overall objective is to identify a promising antiviral agent for ZIKV by conducting a more in-depth antiviral investigation into the effectiveness of FAV against ZIKV in different human tissues.

Higher-dose clinical FAV regimens used to treat patients infected with Ebola reached exposures equivalent to static concentrations of 390 μM [39]; thus, our results indicate that FAV holds potential as an antiviral strategy against ZIKV since therapeutically feasible drug concentrations curbed viral replication in all three cell lines evaluated. These findings are in agreement with previous reports of FAV’s effectiveness against ZIKV [28,32] and other members of the family flaviviridae, such as West Nile virus (WNV) and hepatitis C virus (HCV) [15,40,41]. These studies also mirror our prior findings which showed that the antiviral effect varies considerably depending on the host cell line, as FAV is much more potent in HeLa and HUH-7 cells compared to SK-N-MC cells, thereby illustrating the importance of using multiple cell types when considering the antiviral potential of an agent.

One of the more interesting findings in this work was that in addition to inhibiting ZIKV replication in a dose-dependent manner, FAV activity was enhanced over the course of therapy in HeLa cells; this effect led to a downward trend in infectious titers over time that was not caused by drug toxicity and was absent in SK-N-MC and HUH-7 cells. This is an atypical finding, as usually, effective antivirals will either result in blunted viral titers or a delay in viral replication kinetics. In either scenario, we do not normally observe a decrease in viral burden over time from drug therapy. Therefore, we aimed to evaluate the mechanism of action of FAV in HeLa cells to determine why the antiviral effect was more pronounced in this cell line. By understanding the mechanism of action of FAV in different cell types, we hypothesized that we could better predict FAV effectiveness or better utilize this agent in an effort to maximize viral inhibition.

The examination of the FAV effect on viability of infected cells showed two outcomes: (1) cell health and metabolism of HeLa cells was most negatively affected by ZIKV infection when compared to the other cell types; (2) increasing concentrations of FAV caused vast improvements in the viability of infected HeLa cells. These results suggest that FAV protects HeLa cells (but not SK-N-MC or HUH-7 cells) from a productive viral infection. This finding was surprising since FAV’s proposed mechanisms of action occur later in the viral replication cycle, resulting in either chain termination or lethal mutagenesis of the nascent viral RNA strand [14,15]. Thus, we would not expect treatment to prevent or protect cells from becoming infected. This type of antiviral profile is more consistent with compounds such as interferon that stimulate the host cell antiviral response to make cells refractory to infection or entry inhibitors that prevent viral attachment to host cells [42]. We postulated that the disparity in response to FAV treatment in these three cell lines indicates that FAV’s protective effect in HeLa cells is a main driver in FAV’s robust anti-ZIKV activity in this cell line.

We next investigated how FAV exerted its protective effect against a cytopathic ZIKV infection in HeLa cells. We compared levels of infectious virus and viral genetic material to determine whether FAV treatment protected HeLa cells from ZIKV infection by: (1) hindering production of virus particles, thus lowering the overall amount of virus available to infect cells; or (2) altering viral infectivity and reducing the amount of replication-competent virus released from infected cells. Our results clearly showed that FAV exposure caused marked declines in the amount of infectious virus that could be quantified in this cell line, but RNA levels remained high, even as the concentration of FAV increased. These findings suggest that drug-induced reductions in viral infectivity contributed to FAV’s enhanced effect against ZIKV in HeLa cells. It is important to note that unprotected viral RNA undergoes rapid degradation in tissue culture medium (Figure 6). Therefore, measured RNA levels in our assays correspond to viral RNA that was protected from deterioration, likely by being packaged into virus particles. These results suggest that viral particles continue to be produced in abundant quantities in the presence of FAV, but that a fraction of the viruses released from the cell are not capable of eliciting a productive infection.

These data led us to hypothesize that FAV exerts its antiviral effect on HeLa cells by inducing production of defective viral particles. Defective viral particles are a non-infectious viral particle containing truncated or altered genomes that render them unable to carry out a replication cycle on their own [43,44]. Defective viral particles have been readily described for influenza virus and many other RNA viruses [43,45,46]. These non-infectious viral particles interfere with the replication of infectious virus by competing with replication-competent virus for binding to host cell receptors as well as viral and cellular proteins required for replication, and their accumulation can lead to viral extinction [45,47]. FAV-induced reductions in infectious viral titers through increased production of defective interfering virus particles may explain the observed protective effect in the cell health and metabolism of ZIKV-infected HeLa cells. This hypothesis will be further assessed in a companion paper [48].

Although FAV exposure did not cause the same antiviral effect in SK-N-MC and HUH-7 cells that was observed in HeLa cells, our results demonstrated that treatment with FAV led to slight downturns in viral infectivity in these cell lines. In our companion study, we will also investigate whether our hypothesized mechanism of action in HeLa cells explains the results obtained from studies conducted in SK-N-MC and HUH-7 cells [48].

Virologic outcomes obtained from HeLa cells demonstrated that all evaluated FAV concentrations caused a disparity between levels of infectious virus and viral RNA; however, declines in infectivity were most pronounced at concentrations ≥ 250 μM. This finding suggests that in order to maximize FAV effect in this cell line, drug exposures should ideally be maintained at or above this level. Our experiments also indicated that FAV’s influence on viral infectivity can be reversed following the removal of drug pressure (Figure 7C,D), allowing for the production and release of fully competent viruses in the absence of drug pressure. These findings highlight the importance of selecting dosages that will lead to sustained declines in infectivity over the duration of therapy, since it can be reasoned that a drop in drug exposures below therapeutic levels can markedly curb or completely abolish antiviral effect.

Despite the promising results observed in HeLa cells, one limitation of this study is that our assays were conducted using static drug concentrations. In future studies, we will use the hollow fiber infection model to simulate FAV’s human pharmacokinetic profile in order to determine whether clinical FAV exposures are sufficiently high to appreciably alter viral infectivity over the entire dosing interval, or whether dose adjustments are required to prevent the loss of FAV’s infectivity effect as drug concentrations wane over time.

In this work, we have demonstrated that therapeutically achievable FAV concentrations effectively inhibited the replication of ZIKV in three human-derived cell lines. Additionally, we determined that FAV’s potent antiviral effect in HeLa cells is likely attributed to drug-induced deterioration of viral infectivity. Lastly, we have proposed that FAV’s inhibitory activity is mediated through the production of non-infectious defective viral particles. Our companion study will focus on assessing whether FAV does indeed act as an inducer of defective particles and characterizing the host cell factors that contribute to tissue-specific differences in susceptibility to FAV effect [48].

## Data Availability

Not applicable.
